# Herbaceous Plants as a Phytoremediation Tool in Urban Areas: A Review

**DOI:** 10.3390/plants15111609

**Published:** 2026-05-24

**Authors:** Giulia Nuscis, Emma Cocco, Eleonora Buoio, Jessica Frigerio, Andrea Maxia, Paolo Colleo, Antonio De Agostini, Pierluigi Cortis

**Affiliations:** 1 Department of Life and Environmental Sciences, University of Cagliari, Via S. Ignazio da Laconi 13, 09123 Cagliari, Italypierluigi.cortis@unica.it (P.C.); 2 Department of Veterinary Medicine and Animal Science (DIVAS), Università degli Studi di Milano, Via dell’Università 6, 26900 Lodi, Italy; 3 Department of Biotechnology and Biosciences, University of Milano-Bicocca, Piazza della Scienza 2, 20126 Milano, Italy; jessica.frigerio@unimib.it; 4 Agricultural Research Agency of Sardinia (AGRIS), Viale Trieste 111, 09123 Cagliari, Italy

**Keywords:** herbaceous plant, phytoremediation, heavy metal, urban area, phytoextraction, phytostabilisation

## Abstract

Rising global temperatures, increasing frequency and intensity of extreme climatic events, with associated growth of agricultural land use and urban expansion, represent critical drivers of biodiversity loss. Within this framework, urban areas are particularly vulnerable due to environmental stressors such as the heat-island phenomenon, soil sealing and depletion, and the accumulation of heavy metals and other pollutants. Recent sustainability-oriented urban policies recognize the strategic role of green infrastructures in mitigating these impacts by delivering essential ecosystem services, including phytoremediation. Here, the focus on herbaceous plants allows the selection of species with short life cycles and high colonization rates in marginal or disturbed urban habitats (e.g., roadside verges, compacted soils, and limited-volume planting areas). Therefore, the present review systematically examines herbaceous plant species with documented phytoremediation capabilities, focusing on Mediterranean native taxa evaluated under urban or peri-urban conditions. A total of 29 species met the selection criteria: taxonomically, Asteraceae represented the most frequent family (35%), followed by Fabaceae (21%), Brassicaceae, and Poaceae (each accounting for 10%). From a functional-trait perspective, hemicryptophytes dominated the dataset (66%), followed by therophytes (31%). Of the selected taxa, 55% primarily exhibited phytoextraction, 14% showed phytostabilization, and 31% demonstrated dual functionality, through combined extraction and stabilization pathways. These traits, combined with ecological adaptability to Mediterranean climatic regimes, support their application in Mediterranean urban environments.

## 1. Introduction

Urban green spaces play a crucial role in conserving biodiversity and enhancing human well-being, thereby necessitating strategic planning to expand and improve their quality in response to the increase in the urban population and the resulting landscape transformations [1,2]. Green infrastructures include parks, roadside vegetation, and spontaneous ruderal plant communities, which help maintain ecological balance and provide essential ecosystem services [3,4]. However, in continuously expanding Mediterranean European cities, the conservation and development of the ecosystem services provided by urban green spaces face significant challenges. Major issues include the impact of climate change [5], water resource scarcity, reduced biodiversity within urban areas, limited availability of green spaces [1], and the environmental impact of urban infrastructure [2].

Among the crucial ecosystem services it provides, urban green infrastructure contributes to the mitigation of environmental stressors by promoting nutrient cycling, enhancing primary production, filtering pollutants from above- and below-ground environments, and promoting soil health [6,7,8]. In this sense, several studies have investigated the close relationship between grasslands and soil quality, which supports plant productivity, provides habitat for soil organisms and microorganisms, enhances nutrient availability, contributes to pathogen control, and regulates water dynamics [9]. Moreover, the symbiotic interactions between plants and soil microorganisms enhance resistance to various urban pollutants and contaminants, fostering a positive feedback loop that supports the ecosystem [10,11,12]. Urban soils and air are frequently contaminated by heavy metals and particulate matter, mainly arising from industrial emissions, vehicular traffic, and improper waste disposal. According to the EU Ambient Air Quality Directive 2008/50/EC and Italian DPR 155/2010, limits exist for pollutants such as PM10, PM2.5, NO2, lead (Pb), cadmium (Cd), and arsenic (As) to protect human health and ecosystems. However, many urban areas in the Mediterranean region exceed these thresholds [13].

Even at low concentrations, heavy metals can be toxic to plants, animals, and the human population, accumulating in living tissues and causing health issues in humans and negative impacts on ecosystems [14]. From a plant perspective, certain metals such as iron (Fe), zinc (Zn), copper (Cu), and nickel (Ni) are essential micronutrients that play crucial roles in cellular and metabolic processes, although they become toxic when present in excess. In contrast, heavy metals including cadmium (Cd), mercury (Hg), silver (Ag), lead (Pb), and chromium (Cr) have no known biological function and exhibit toxicity even at low concentrations [6,15].

Pollution of urban areas poses serious risks to the environment and human health, highlighting the need for innovative and sustainable solutions. Among them, phytoremediation has emerged as a promising ecological and biotechnological approach to mitigate soil and air contamination. Phytoremediation identifies several plant-based actions aiming at the uptake, accumulation, and detoxification of heavy metals in contaminated environments. The mechanisms by which phytoremediation delineates include phytoextraction, phytostabilization, phytovolatilization, and rhizofiltration. Phytoextraction involves the absorption of metals from the soil and their accumulation in plant tissues, primarily in leaves and stems, enabling the subsequent harvesting and removal of the contaminants [15]. This plant’s tolerance strategy relies on the detoxification of heavy metals to minimize their impact on cellular functions, mainly through chelation, in which free heavy metal ions are complexed with ligands, reducing their concentration to relatively low levels [16,17]. In contrast, phytostabilization immobilizes pollutants within the soil, reducing their bioavailability through root exudates and interactions with the soil matrix [12]. Some plants, through phytovolatilization, transform certain metals into less toxic gaseous forms, while rhizofiltration helps to filter contaminants from water systems [18].

Recent years have seen growing interest in heavy metal-tolerant plants due to their potential applications in phytoremediation and reclamation of contaminated sites. These plants have evolved specialized physiological and biochemical mechanisms that enable the effective sequestration of metals within various cellular compartments, allowing them to tolerate, absorb, and accumulate high concentrations of metals in their tissues, particularly in leaves and stems [19].

In this context, herbaceous plants, classified as non-woody species characterized by soft, flexible stems and the absence of persistent above-ground structures, demonstrate remarkable potential for metal tolerance and accumulation due to their adaptability, rapid growth rates, and morphological plasticity [15,18,20,21]. In urban environments, their characteristics offer valuable opportunities to employ herbaceous plants in mitigating the ecological and biodiversity disruptions commonly associated with pollution in large European cities. Several studies have, in fact, demonstrated that certain spontaneous herbaceous plants exhibit high tolerance to heavy metals in contaminated areas and can adapt well to harsh environmental conditions, highlighting their potential utility in phytoremediation processes [3,22]. These investigations have been conducted in various regions worldwide, including Mediterranean areas, where wild herbaceous species have been observed and analyzed while colonizing environments contaminated by heavy metals, such as abandoned mine sites [23,24], large urban areas degraded by anthropogenic activities, roadsides, and agricultural lots [15,25,26].

Despite the nutrient-poor status of polluted soils, many native herbaceous species have shown success in colonizing these areas by developing mechanisms of tolerance and resistance. Moreover, the use of native and adaptive plant species in green infrastructure projects ensures long-term environmental sustainability while simultaneously reducing maintenance costs, particularly in terms of irrigation and fertilization requirements [10,13,27]. A combination of ecological, molecular, and soil management approaches can significantly enhance the effectiveness of phytoremediation, contributing to recreating cleaner and healthier urban environments.

Future research should focus on quantifying the phytoremediation efficiency of these species under field conditions, optimizing plant–microorganism interactions, and developing policy frameworks to incorporate phytoremediation into urban planning, ultimately converting contaminated spaces into multifunctional green assets. The aim of the review is to identify native Mediterranean herbaceous species with proven tolerance and/or accumulation of relevant heavy metals (such as lead “Pb”, cadmium “Cd”, zinc “Zn”, copper “Cu”, arsenic “As”, mercury “Hg”, nickel “Ni”, chromium “Cr”) in urban environments, emphasizing their potential to integrate multifunctional greening solutions in degraded urban contexts.

### Urban Soil Contamination in the Mediterranean Context

To better contextualize the challenges of soil contamination and the potential of native herbaceous flora, Italy is hereafter presented as a representative case study for the Mediterranean basin. Due to its central geographical position, diverse phytoclimatic regions, and long history of industrial and mining activities, the Italian landscape encapsulates the broader environmental dynamics observed across Southern Europe. Numerous investigations carried out in various Italian cities revealed widespread heavy metal contamination in urban soils, with concentrations often exceeding regulatory limits established for residential and green areas. Below, we provide a brief overview of the most relevant results documented in recent years [28,29,30,31,32,33,34].

In the Campania region, early studies conducted in the urban area of Naples [30,31,32,33,34,35] revealed high concentrations of Cu, Pb, and Zn in soils surrounding industrial facilities and roadways. In 2003, Imperato et al. [30] divided the urban area into regular grids and sampled gardens, parks, roadside zones, and industrial sites, including both the western and eastern districts of the city affected by the presence of a major highway and former refineries. The analyses showed a substantial spatial variability in Pb concentrations, with significant peaks near highways and high-traffic roads. Vehicular emissions were identified as the main source of Pb, while Zn and Cu were attributed to tire and rail abrasion, as well as residual contamination from previous industrial activities. Compared to 1974 data, concentrations of Cu, Pb, and Zn had increased, particularly Zn in roadside soils, indicating a progressive rise in atmospheric deposition from traffic over time.

In Sicily, a study conducted in the city of Palermo found elevated levels of Pb, Zn, Cu, Sb, and Hg in parks and green areas exposed to heavy traffic, highlighting the significant role of vehicular emissions [31].

In Marche, analysis carried out in the coastal city of Ancona revealed that more than half of the 18 sampled sites exceeded the regulatory thresholds for multiple metals (especially Pb and Zn) attributed to the combined effects of road, rail, and maritime traffic, as well as commercial activities [36].

In Tuscany, the soils of Fornaci di Barga were found to be contaminated with Cu, Zn, and Cd, even in sensitive areas such as school gardens and public parks, due to metallurgical activities and vehicular traffic [37]. In Pisa, contamination is less concentrated, except for certain sites enriched in Zn and Hg—the latter potentially resulting from illegal disposal of industrial waste [29].

In Piedmont, the metropolitan area of Turin presents a complex situation in terms of soil quality. Within this area, the municipality of Grugliasco showed soil samples exceeding regulatory thresholds for Ni, Zn, and Pb, with contamination sources primarily linked to anthropogenic activities such as vehicular traffic, foundries, lubricants, and waste incinerators [33]. A study conducted along a 7.5 km urban transect revealed elevated concentrations of Cu, Cr, Ni, Pb, and Zn, particularly near roundabouts and traffic lights, where high traffic intensity promotes metal accumulation in soils. For Ni and Cr, a geogenic component was also observed, attributed to the weathering of serpentinite-rich substrates in alluvial deposits [32]. Data collected by Yan et al. [34] confirmed a spatial contamination gradient, with increasing values from the peri-urban areas toward the city center, where the highest concentrations were recorded. Similarly, Biasioli and Ajmone-Marsan [28] reported elevated contamination levels in urban soils of Turin, particularly in public parks. Although these areas are primarily subject to diffuse contamination, metal concentrations (Co, Cr, Ni, Pb, Sn, and Zn) frequently exceeded regulatory thresholds. Pb, Cu, and Zn were closely associated with urban traffic: lead historically from leaded gasoline combustion, copper from brake wear, and zinc from tire degradation. Additional sources include emissions from industrial plants and incinerators, confirming the multifactorial nature of urban soil pollution in Turin.

Overall, these studies demonstrate a clear positive correlation between land use, urban density, traffic, and industrial activities with increasing heavy metal pollution in urban soils. The most frequently detected metals include Cu, Zn, Pb, Cd, Ni, and, in some cases, Hg and Cr. These elements are strongly associated with anthropogenic activities, particularly transportation, metallurgical industries, and improper waste management. Heavy metal pollution in Italian urban soils is therefore widespread and influenced by both historical and ongoing contamination sources. Therefore, the evidence gathered from the Italian context serves as a proxy to understand the broader potential of Mediterranean herbaceous species in mitigating soil pollution throughout the basin.

## 2. Results

### 2.1. Mediterranean Herbaceous Plants for Urban Remediation

Many studies on tolerant herbaceous species capable of accumulating heavy metals have emphasized their potential role in urban environments, particularly by highlighting their adaptive strategies and their ability to thrive in anthropogenically disturbed areas. In the present study, a systematic literature review was conducted to select research carried out specifically in Mediterranean urban environments, with particular attention to studies demonstrating the phytoremediation capacity of herbaceous plants to tolerate heavy metals. Research has been conducted in various Mediterranean cities, including several sites in Italy, with a special focus on environments contaminated or degraded by anthropogenic pressures. According to these criteria, a total of 30 articles were identified and included in the analysis.

**Figure 1:**
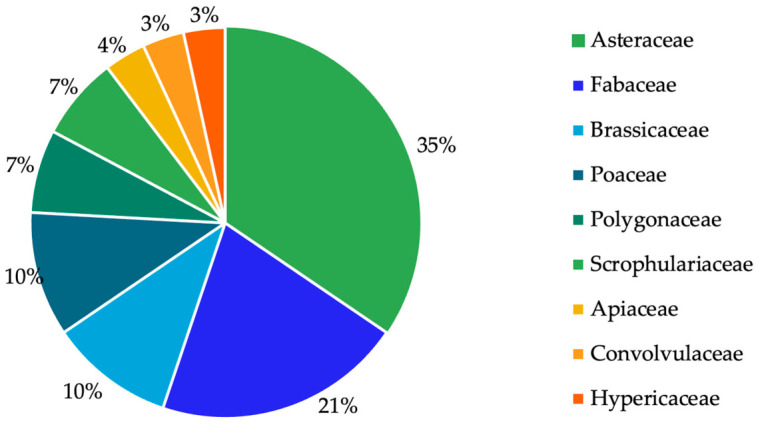
Percentage of species meeting the inclusion criteria—namely, high tolerance and accumulation of heavy metals, ability to grow in urban and polluted environments, and belonging to the autochthonous Mediterranean flora—divided by plant family.

Through the literature review approach implemented in the present study, several plant families were identified as being featured by high tolerance and strong accumulation capacity of heavy metals, making them particularly suitable for the remediation of polluted soils and degraded environments. Among the herbaceous plant families that best matched the selected criteria—namely, tolerance and accumulation of heavy metals, ability to grow in urban and polluted environments, and belonging to typical Mediterranean flora—Asteraceae, Fabaceae, Brassicaceae, and Poaceae emerged as the most representative (Figure 1).

### 2.2. Ecological Strategies of Dominant Families

According to Baldi et al. [38], the frequent occurrence of species belonging to Asteraceae and Poaceae in the disturbed contexts of interest can be attributed to shared ecological strategies, such as high reproductive efficiency and strong resistance to environmental stress: Asteraceae benefit from wind-dispersed seeds (facilitated by the presence of a pappus), and tolerate trampling due to their rosette leaf growth form, while many Poaceae are perennial weeds that spread rapidly through vegetative growth and exhibit significant trampling resistance thanks to the basal position of their apical meristems. However, given the selection process applied in this study, many Poaceae species were not included in the selected species. This is due to their often invasive behavior, low floral attractiveness to pollinators (as a result of anemophilous pollination syndrome), and limited ornamental value. Moreover, their anemophilous reproductive strategy, involving the release of large quantities of wind-dispersed pollen, is commonly associated with allergenic responses [39].

### 2.3. Life Forms and Urban Adaptations

**Figure 2:**
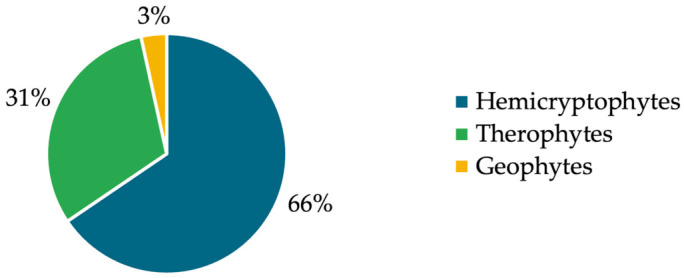
Number of species and percent presence of selected species divided by life forms.

From the point of view of the biological spectra, based on the Raunkiær system, the majority of the taxa identified within the selected families belong to the hemicryptophyte life form, followed by therophytes, and to a lesser extent, geophytes (Figure 2). This distribution pattern is likely influenced by the extreme environmental conditions typical of urban areas, which intensify competition among plant species and tend to favor more opportunistic taxa. In particular, therophytes and many hemicryptophytes species are characterized by short life cycles and rapid adaptive strategies, which confer a selective advantage under these conditions [38]. Both life forms are well-suited to urban environments and are prominently represented within the Mediterranean herbaceous flora [40].

### 2.4. Phytoremediation Capacity

**Figure 3:**
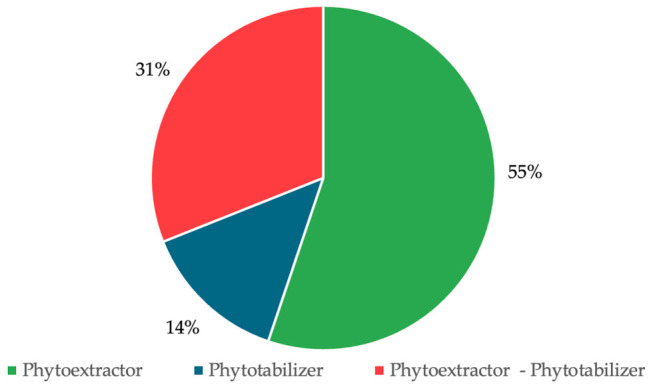
Proportion of phytoextractor, phytostabilizer, and phytoextractor–phytostabilizer entities among the selected species.

**Figure 4:**
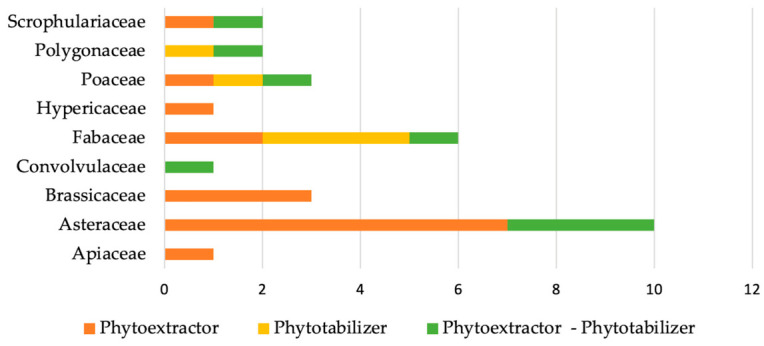
The chart shows the distribution of phytoextractors, phytostabilizers, and phytoextractor–phytostabilizer species among families. Phytoextractor is represented in green, phytostabilizer in blue, and phytoextractor–phytostabilizer in yellow.

Most of the selected species exhibit marked phytoextraction capacity (Figure 3 and Figure 4), making them particularly suitable for the remediation of contaminated soils. This factor, together with their ecological adaptability and resilience towards environmental stressors, underscores their relevance in the selection of plant species to be integrated in urban phytoremediation projects.

### 2.5. Herbaceous Plants Suitable for Phytoremediation

Following a thorough analysis of the scientific literature, several plant species exhibiting considerable heavy metal accumulation and tolerance capacities have been identified (Table 1). These species, belonging to various families and distinguished by their adaptability to contaminated soils, are to be considered for potential use in phytoremediation approaches—namely phytoextraction and phytostabilization—thereby contributing to environmental remediation in urban areas.

## 3. Discussion

### 3.1. Asteraceae

Within urban green infrastructures, Asteraceae Martinov are among the most widespread plant families, notable for their capacity to accumulate and tolerate heavy metals. *Dittrichia viscosa* L. Greuter is a common melliferous species often found in ruderal and urban habitats. In the study conducted by Guarino et al. [25], at the Bagnoli-Coroglio National Interest Site (SIN), characterized by multi-element contamination, both soil and plant tissue samples were analyzed to evaluate heavy metal accumulation and phytostabilization capacity of various plants. Chemical analyses revealed that *D. viscosa* concentrates Cu and thallium (Tl) in its roots, indicating its potential for both phytostabilization and phytoextraction. This capacity has also been observed in studies conducted in mining areas, which highlighted the ability of *D. viscosa* to accumulate high concentrations of As, Cd, Pb, and Zn [3,48]. Another noteworthy species is *Taraxacum sect. Taraxacum* F.H. Wigg, commonly known as dandelion, is recognized as a bioindicator and phytoremediator of heavy metals in urban environments. This species, which thrives even in small urban spaces and degraded areas, can accumulate high concentrations of Pb, Zn, and Cu with seasonal variability in its distribution. According to Giacomino et al. [54], the accumulation capacity of dandelion follows the order Cd > Zn > Cu > manganese (Mn) > Pb ≅ Fe ≅ Cr. This confirms the pronounced and well-documented ability of *T. sect. Taraxacum* to accumulate Cu, Zn, and particularly Cd from its surrounding environment. Several studies reported that metal concentrations are typically higher in the roots than in the leaves; however, in urban contexts, atmospheric deposition significantly contributes to increased metal concentrations in the foliage [10,13,22]. In addition to its phytoremediation potential, *T. sect. Taraxacum e* plays a crucial role in the urban ecosystem as a melliferous species, providing nectar and pollen resources to pollinators, particularly bees [55]. Thanks to these characteristics, it is considered a valuable environmental pollution bioindicator and a potential phytoremediator. *Cichorium intybus* L., commonly known as chicory, grows in uncultivated and/or low-productivity soils, ruderal and natural habitats, as well as in urban gardens [10,60]. Its capacity to absorb and accumulate heavy metals has been documented by Pietrelli et al. [26] in various environments, including both urban and rural areas within the city of Rome. Results showed that chicory exhibits a high accumulation capacity, with Cd primarily concentrated in flowers, Zn and Cu in leaves, and Pb in roots [47]. Additionally, Cd, Cr, Cu, Ni, Pb, and Zn have been recorded in shoots [42], confirming their potential as phytoremediators. Metal uptake by chicory varies according to soil composition and element availability. In urban sites, where contamination levels are higher, heavy metal accumulation in plants is more pronounced compared to those grown in rural areas [26]. Moreover, chicory holds significant importance due to its ornamental value and its ability to attract insects [10]. *Picris hieracioides* L. is a widespread species capable of adapting to degraded and contaminated soils, promoting their remediation through its tolerance and accumulation of heavy metals [51,52]. A recent study by Pietrelli et al. [26], conducted in Rome, revealed its capacity to accumulate metals in various organs with differentiated distribution: Pb and Zn across all tissues, elevated Cu concentrations in basal and cauline leaves, and Cd accumulation in flowers. *Hypochaeris radicata* L., a common species in Italian urban areas [1,60], has been extensively studied in different disturbed soil types. According to Bretzel et al. [10], it holds an ornamental role due to its flowers, which attract pollinators and contribute to urban biodiversity. Research on its phytoremediation and metal accumulation processes has demonstrated its ability to tolerate and absorb various heavy metals, including Cr, S, Ni, Mn, and Fe [44]. More recent studies have further identified its capacity to accumulate uranium (U) and Zn in roots and leaves, reinforcing its suitability in bioremediation applications within contaminated environments [14,49,50]. *Achillea ageratum* L., *A. millefolium* L.—and the subspecies *A. millefolium* L. subsp. *millefolium*—are herbaceous plants known for their ability to absorb and accumulate heavy metals. However, studies regarding their potential use in phytoremediation remain limited and largely outdated. *A. millefolium* shows a specific tendency to accumulate Zn in shoots [42], while its subspecies has been reported to accumulate Fe, Cr, Mn, and S in aerial parts [44]. The high tolerance and growth capacity in contaminated soils make *A. millefolium* a promising candidate for phytoremediation strategies [43]. Additionally, *A. ageratum* L. has demonstrated the ability to accumulate antimony (Sb) [41], broadening interest in the genus *Achillea L.* for phytoremediation of contaminated soils. Several studies have also explored the capabilities of both woody and herbaceous ornamental plants for phytoremediation in urban and peri-urban environments [15,21]. Among the herbaceous ornamental plants discussed, it should be noted that certain taxa do not exhibit a uniform native status across the entire Mediterranean basin, being indigenous only to specific countries or sub-regions. In such cases, careful consideration is required for practical field applications to avoid the introduction of non-native genotypes. A prominent example within the Asteraceae family is *Calendula officinalis* L.; although widely naturalized, it is strictly native to the Iberian Peninsula (Spain) [61]. Nonetheless, this species remains highly relevant for phytoremediation due to its dual role in enhancing soil quality through contaminant absorption and providing esthetic value to the urban landscape [60,62]. Regarding its phytoremediation potential, *C. officinalis* accumulates Cd, translocates Cr and Cu, and tolerates high concentrations of Pb. Notably, this species also shows significant accumulation of Cu and Pb in roots and leaves, making it an excellent candidate for the phytoremediation of polluted soils. Furthermore, it can adapt to soils with low Cr concentrations, confirming its adaptability in addressing different types of contamination. *Helianthus annuus*, commonly known as the sunflower, is capable of translocating various heavy metals such as Cu, Cd, and Ni, with a high accumulation capacity of Cd and Ni observed in inflorescences. It is reported to effectively remove Cu, Cr, and Zn from contaminated soils and accumulate Pb [50]. Moreover, its capacity to absorb high levels of As can be significantly enhanced through the use of soil amendments or fertilizer [19,20,21,42,45]. Raklami et al. [46] report that the addition of bacterial inoculants improves *H. annuus* phytoextraction of cobalt (Co), Pb, and Zn. Similarly to *H. annuus*, *C. officinalis* has great potential for the phytoremediation of As-contaminated soils [21,45]. Both plants exhibit valuable traits for phytoremediation techniques such as phytoextraction and phytostabilization of heavy metals, due to their ability to accumulate and tolerate metals within environmental remediation contexts.

### 3.2. Fabaceae

Numerous studies highlight the ability of many species within the Fabaceae Lindl. family to tolerate and accumulate heavy metals. Among these, *Lotus corniculatus* L. is a common herbaceous species in Italy that thrives in a wide range of soil conditions, including compact, dry, moist, acidic, saline, and nutrient-poor substrates. In the study conducted by Guarino et al. [25], this species demonstrated a high accumulation capacity for Pb, V, and As, confirming its usefulness for phytoremediation, consistent with findings from previous studies [20,22,24]. *Medicago lupulina* L. appears more suitable in phytostabilization of metals in soil, due to its limited translocation capacity to the aerial parts [20,25]. According to Massa et al. [22], in a study examining the vegetation from one of the 15 critical environmental sites in Italy, a waste dump of the chemical factory ACNA, Aziende Chimiche Nazionali Associate—Associated National Chemical Companies, both *L. corniculatus* and *M. lupulina* displayed high photosynthetic efficiency, indicating good tolerance to multiple heavy metals. Other Fabaceae species, such as *Trifolium repens* L., *T. arvense* L. var. *arvense*, *Lupinus albus* L., and *Anthyllis vulneraria* L. have shown the ability to simultaneously tolerate and accumulate several heavy metals without evident signs of physiological stress, suggesting strong phytoremediation potential. *T. repens* appears to be an effective phytostabilizer for Cd and Pb [16]. *A. vulneraria* is a drought-tolerant species well-suited for phytostabilization in environments affected by polymetallic and metalloid contamination, tolerating high concentrations of As, Cu, Pb, and Zn [3,14,22,42,44]. In addition to their phytoremediation capabilities, *L. corniculatus* and *A. vulneraria* also possess ornamental value and are well-known for their ecological importance and melliferous properties, contributing to biodiversity and supporting pollinating insects [5,63].

### 3.3. Brassicaceae

Brassicaceae Burnett are among the primary families of heavy metal-tolerant and hyperaccumulator plants, with high potential for phytoextraction [14,25,53]. This family includes several herbaceous species capable of tolerating and accumulating significant quantities of heavy metals. A notable example is *Hirschfeldia incana* (L.) Lagr.-Foss., which has demonstrated the ability to tolerate and accumulate Cu and Tl, hyperaccumulate Pb [3,20], and accumulate Zn in both shoots and roots [49]. *Brassica napus* L. is particularly effective in accumulating Cr, and when inoculated with plant growth-promoting rhizobacteria (PGPR), it exhibits enhanced biomass production as well as improved uptake and translocation of Cd to the leaves [20,46]. *B. rapa* L. also shows a strong capacity for absorbing Cd, Fe, and Zn [20]. These species are common in Mediterranean regions and have developed adaptive strategies to withstand environmental stressors such as drought and high ion concentrations. In the Italian territory, the latter species, *B. napus* and *B. rapa,* are present as alien species, either casual or naturalized [64], and are typically found colonizing fallow or anthropogenically disturbed soils.

The heavy metal accumulation capacity of herbaceous species belonging to the Scrophulariaceae Juss. family, *Verbascum thapsus* L. [26] and *Verbascum sinuatum* L. [25], has been analyzed in two geographically distinct areas. The first study was conducted in Rome and considered two sites: an urban location near a high-traffic road in the city center and a rural site within the Canale Monterano park, located in the north of Rome. The second study focused on the Bagnoli-Coroglio SIN area (Site of National Interest), located in the western part of Naples and characterized by severe and complex contamination. Both species demonstrated good tolerance and effective accumulation of several heavy metals. *V. thapsus*, collected in the urban site in Rome, showed notable accumulation of Pb, with the highest concentrations recorded in the leaves and stems, while Cu and Zn were more evenly distributed across all plant organs. The dense pubescence of *V. thapsus* leaves appears to enhance the capture and retention of airborne pollutants [26]. *V. sinuatum* exhibited a pronounced capacity to accumulate Cu in the roots, showcasing the highest concentration among all analyzed species [25]. This species was thus identified as a promising candidate for Cu and also Hg phytoextraction, with moderate Pb translocation. It also showed limited translocation capacity for V, indicating that this element remains primarily confined to the root system [25]. In summary, *V. thapsus* appears to be more effective in Pb accumulation (leaves and stems), while *V. sinuatum* is a good Cu accumulator (roots) and shows moderate capacity for V uptake. Both species demonstrate good heavy metal tolerance yet differ in their metal-specific accumulation and translocation patterns.

### 3.4. Poaceae and Other Families

*Piptatherum miliaceum* L. Coss., *Festuca rubra* L., and *F. arundinacea* L. are perennial herbaceous species belonging to the Poaceae (R.Br.) Barnhart family.

*F. rubra* has been widely reported as a metal-tolerant and metal-accumulating species. In particular, the study conducted by Fernández et al. [24] highlighted its capacity to thrive in highly polluted soils, including those enriched with Pb-Zn or Hg-As, accumulating primarily Zn or Hg in the aerial parts depending on the specific soil contamination. Additional studies have confirmed its ability to accumulate Fe [22] and Cu [20]. According to Sladkovska et al. [16], *F. rubra* is also effective in phytostabilizing Zn, Cd, Pb, and Cu. Similarly, *P. miliaceum* has demonstrated tolerance to soils contaminated with Pb-Zn or Hg-As, showing the ability to accumulate Zn and Hg in its aerial tissues [20,24]. In contrast, this species tends to retain Cu and Cd mainly in the roots, confirming a moderate yet effective phytostabilization potential [25], which was further supported by more recent findings [50]. *F. arundinacea*, as indicated by Guarino et al. [25], stands out as one of the most promising candidates for phytoextraction, due to its ability to accumulate As in the roots and Cd in the aerial parts. Sladkovska et al. [16] also emphasize its potential for use in the remediation of contaminated soils, particularly for the uptake of Zn and Pb.

*Daucus carota* L., a member of the Apiaceae Lindl. family, is widespread in degraded urban environments. Being characterized by finely divided foliage and compound umbels bearing small white or yellowish flowers, this wild herbaceous species plays an important ecological role as a nectar source for pollinators [55,63]. Studies on its bioaccumulation potential have demonstrated the presence of heavy metals in various plant organs, suggesting its suitability as a tool for environmental monitoring and phytoremediation. Fernández et al. [24] reported that *D. carota* can thrive in alkaline mining soils rich in Hg and As, with a strong tendency to hyperaccumulate Hg in the aerial parts. According to Massa et al. [22], D. carota growing at the ACNA industrial SIN site on the waste dump “A5”, accumulated significant amounts of Fe in both shoots and roots. Pietrelli et al. [26] further found that the distribution of heavy metals in D. carota varies across plant organs and is influenced by soil properties: flowers tend to accumulate Pb and Cu, roots show higher concentrations of Pb and Zn, leaves exhibit notable levels of Cd, Cu, Ni, Pb, and especially Zn, while stems mainly accumulate Cu and Zn. Their findings also revealed that the plant’s capacity to translocate metals to aerial tissues is enhanced in highly contaminated urban soils.

*Hypericum perforatum* L. (Hypericaceae Juss.), commonly known as St. John’s wort, is a perennial herbaceous plant characterized by yellow flowers and punctate leaves. It adapts well to a variety of environments and demonstrates promising phytoremediation potential. This species exhibits a notable tolerance to Cr, which is primarily accumulated in flowers [14,46], where high concentrations of Ni have also been detected. Additionally, it shows a significant capacity to absorb Pb, concentrating it mainly in the roots and, to a lesser extent, in the aerial parts. This accumulation pattern may be associated with the presence of opercula on the leaves, which may facilitate metal uptake [26].

*Convolvulus arvensis* L. (Convolvulaceae Juss.) is a widespread species found in various environments, including fallow lands, meadows, roadsides, gardens, and anthropogenic habitats. In urban areas, it plays an important ecological role as a nectar source for pollinators [55]. Based on the high concentrations of several heavy metals detected in different plant organs in multiple chemical analyses [22,55], *C. arvensis* has demonstrated the ability to both tolerate and accumulate metals present in the soil. Massa et al. [22] observed, through experimental results, a good photosynthetic efficiency indicative of its metal tolerance alongside the presence of seven heavy metals primarily in the shoots and, to a lesser extent, in the roots. Notably, this species is capable of hyperaccumulating Cu, Cr, and Fe [20].

*Polygonum aviculare* L. subsp. *aviculare* (Polygonaceae Juss.) has been identified as a heavy metal accumulator species, exhibiting a strong growth capacity even in highly disturbed environments. It is commonly found in trampled areas, along roadsides, in anthropogenic habitats, and in fallow fields, where it can form extensive colonies [58]. Based on biomass analysis, *P. aviculare* is capable of absorbing various heavy metals, including Cd, Cr, Cu, Ni, Pb, Fe, and Zn [58,59]. Furthermore, the species displays a notable tendency to hyperaccumulate Hg, particularly in the shoots and roots. These characteristics make *P. aviculare* a potentially effective candidate for phytostabilization processes. However, while its limited biomass production may hinder large-scale applicability in phytoremediation programs, it remains suitable to be included in greening projects in urban areas [20,22]. *P. arenastrum* Boreau is a species typical of anthropogenic and heavily trampled habitats [64], showing high tolerance to heavy metals contaminated soils. Concentrations of Cr, Cu, Fe, Pb, and Zn in both shoots and roots positively correlate with total and bioavailable metal levels in the soil. This species exhibits a capacity to accumulate Cr even under low-contamination conditions, with the highest Cr bioaccumulation occurring in the shoots. It also shows strong translocation capacity for cobalt (Co) and Pb, regardless of the site’s pollution level. Despite concentrations of Cu, Pb, and Zn in plant tissues at levels considered phytotoxic for other species, *P. arenastrum* displays no visible phytotoxicity symptoms in the shoots, even under elevated concentrations of F (fluorine) and heavy metals [34,47]. For this reason, *P. arenastrum* is confirmed as a promising candidate for phytoremediation applications.

## 4. Materials and Methods

### 4.1. Search Strategy

A systematic literature review has been conducted using the Google Scholar database, focusing on studies published in recent decades that report environmental (e.g., phytoremediation) and social (e.g., enhancement of ecosystem services) benefits associated with herbaceous plants.

The eligibility criteria for selecting papers for the analysis required that studies specifically address heavy metal tolerance and accumulation in plants. To ensure a comprehensive capture of diverse thematic areas, the literature search was structured using a multi-level clustering approach. The keywords were organized into two primary search clusters: Cluster 1 focused on the phytoremediation potential of plants in contaminated soils, utilizing the following Boolean string: (“phytoremediation” OR “heavy metals” OR “metal accumulation”) AND (“plants” OR “native” OR “spontaneous”) AND (“contaminated soil” OR “mining” OR “urban”). Cluster 2 targeted studies specifically focused on urban environments, using the string: (“heavy metal accumulation” OR “trace metals”) AND “plants” AND (“urban” OR “roadside”). The search was restricted to keywords present in the article titles. This initial search yielded a total of 637 articles. These results underwent a rigorous manual screening process based on specific inclusion/exclusion criteria: (i) geographical area, restricting the scope exclusively to Mediterranean regions; (ii) growth form, focusing solely on herbaceous species; (iii) exclusion of crops and alien species; and (iv) exclusion of non-urban or urbanized habitats. Following this multi-step filtering procedure, the dataset was progressively reduced to 30 articles. This geographical, botanical, and land-use refinement was essential to align the dataset with the core objectives of the review.

### 4.2. Data Extraction and Synthesis

The following information was extracted from the selected contributions: species studied, family, biological form, type of phytoremediation activity, according to the authors’ classification (e.g., phytoextraction, phytostabilization), metals studied, accumulation values (where available), urban context, site, and any ecological interactions (pollinators, resilience).

Heavy metals considered were arsenic (As), cadmium (Cd), chromium (Cr), copper (Cu), iron (Fe), lead (Pb), mercury (Hg), nickel (Ni), vanadium (V), and zinc (Zn).

## 5. Conclusions

This literature analysis highlights the significant phytoremediation potential of various herbaceous plant species, particularly native or naturalized taxa in the Mediterranean region, which demonstrate strong adaptability to urban and peri-urban environments. Representative species from families including Asteraceae, Fabaceae, Brassicaceae, Polygonaceae, and Poaceae demonstrate a wide spectrum of adaptive strategies, enabling them to accumulate, tolerate, or stabilize heavy metals such as Pb, Cd, Zn, Cu, As, and Hg in contaminated soils. Several taxa, including *Dittrichia viscosa*, *Taraxacum sect. Taraxacum* F.H.Wigg, *Festuca rubra*, *Daucus carota*, and *Polygonum aviculare* emerge as particularly effective candidates for phytoremediation interventions, combining high metal uptake with ecological functions like pollinator support, soil stabilization, and landscape esthetic value. The variability in accumulation patterns between plant organs and the influence of site-specific soil properties underline the importance of selecting appropriate species based on local environmental conditions and targeted remediation goals (e.g., phytoextraction vs. phytostabilization). Furthermore, the use of spontaneous and low-maintenance herbaceous species could represent a sustainable and cost-effective approach to urban soil restoration, particularly in post-industrial or heavily anthropized areas. Future research should focus on field-scale validation of these species under different contamination scenarios, the optimization of agronomic practices (e.g., amendment application, microbial inoculation), and the assessment of potential ecological risks associated with biomass disposal. Integrating phytoremediation strategies into urban green infrastructure planning can contribute not only to soil remediation but also to broader ecosystem services and urban sustainability.

## Figures and Tables

**Table 1:** Plant species included in the systematic literature analysis, with taxonomic family, biological form, predominant phytoremediation activity, target metals, and corresponding bibliographic references.

No	Species	Family	Biological Form	Activity	Heavy Metals											References
					Cr	Cu	Zn	Ni	Cd	Pb	Mn	As	Hg	Fe	Sb	
1	*Daucus carota* L.	Apiaceae	H	Phytoex		•	•	•	•	•			•	•		[22,24,26]
2	*Achillea ageratum* L.	Asteraceae	H	Phytoex											•	[41]
3	*Achillea millefolium* L.	Asteraceae	H	Phytoex			•									[42,43]
4	*Achillea millefolium* L. subsp. *millefolium*	Asteraceae	H	Phytoex	•						•			•		[44]
5	*Calendula officinalis* L.	Asteraceae	H–T	Phytoex Phytostab	•	•			•	•		•				[21,45,46]
6	*Cichorium intybus* L.	Asteraceae	H	Phytoex	•	•	•	•	•	•						[26,42,47]
7	*Dittrichia viscosa* (L.) Greuter	Asteraceae	H	Phytoex Phytostab	•	•	•		•	•		•				[3,25,48]
8	*Helianthus annuus* L.	Asteraceae	T	Phytoex Phytostab	•	•	•	•	•	•		•				[19,20,21,42,45,46]
9	*Hypochaeris radicata* L.	Asteraceae	H	Phytoex	•		•	•			•			•		[14,26,44,49,50]
10	*Picris hieracioides* L.	Asteraceae	H	Phytoex		•	•		•	•						[26,51,52]
11	*Taraxacum sect. Taraxacum* F.H.Wigg.	Asteraceae	H	Phytoex	•	•	•		•	•	•			•		[13,22,53,54]
12	*Brassica napus* L.	Brassicaceae	T	Phytoex	•				•							[20,46]
13	*Brassica rapa* L.	Brassicaceae	T	Phytoex			•		•					•		[20]
14	*Hirschfeldia incana* L.	Brassicaceae	H	Phytoex		•	•			•						[3,20,49]
15	*Convolvulus arvensis* L.	Convolvulaceae	G	Phytoex Phytostab	•	•					•			•		[3,20,22,44,55]
16	*Anthyllis vulneraria* L.	Fabaceae	H–T	Phytostab		•	•			•		•				[3]
17	*Lotus corniculatus* L.	Fabaceae	H	Phytoex						•		•				[20,22,24,25]
18	*Lupinus albus* L.	Fabaceae	T	Phytoex		•	•		•	•	•	•				[3,14]
19	*Medicago lupulina* L.	Fabaceae	T	Phytostab						•						[20,22,25]
20	*Trifolium arvense* L. var. *arvense*	Fabaceae	T	Phytoex Phytostab	•	•	•	•	•	•						[42,44]
21	*Trifolium repens* L. subsp. *repens*	Fabaceae	H	Phytostab	•			•	•	•				•		[14,16,22,42,44]
22	*Hypericum perforatum* L.	Hypericaceae	H	Phytoex	•			•		•						[14,26,56]
23	*Festuca arundinacea* Schreb.	Poaceae	H	Phytoex			•		•	•		•				[16,25]
24	*Festuca rubra* L.	Poaceae	H	Phytoex Phytostab		•	•		•	•			•	•		[16,20,22,24]
25	*Piptatherum miliaceum* (L.) Coss.	Poaceae	H	Phytostab		•	•		•				•			[20,24,25,50]
26	*Polygonum arenastrum* Boreau	Polygonaceae	T	Phytoex Phytostab	•	•	•			•	•			•		[44,57]
27	*Polygonum aviculare* L. subsp. *aviculare*	Polygonaceae	T	Phytoex Phytostab	•	•	•	•	•	•			•	•		[20,22,58,59]
28	*Verbascum sinuatum* L.	Scrophulariaceae	H	Phytoex Phytostab		•				•			•			[25]
29	*Verbascum thapsus* L.	Scrophulariaceae	H	Phytoex		•	•			•						[26]

Abbreviations. Biological form: H, Hemicryptophytes; T, Therophytes; G, Geophytes. Phytoremediation activity: phytoextraction (Phytoex) and/or phytostabilization (Phytostab). • indicates the phytoremediation action for the corresponding metal.

## Data Availability

All data supporting the findings of this study are available within the paper.
